# Structural Insights into the Folding Defects of Oncogenic pVHL Lead to Correction of Its Function In Vitro

**DOI:** 10.1371/journal.pone.0066333

**Published:** 2013-06-20

**Authors:** Merav D. Shmueli, Lee Schnaider, Daniel Rosenblum, Gal Herzog, Ehud Gazit, Daniel Segal

**Affiliations:** Department of Molecular Microbiology and Biotechnology, George S. Wise Faculty of Life Sciences, Tel Aviv University, Tel Aviv, Israel; Universite de Sherbrooke, Canada

## Abstract

Loss of function mutations in the von Hippel-Lindau (pVHL) tumor suppressor protein are tumorigenic. *In silico* analysis of the structure and folding of WT pVHL identified in its core an aromatic tetrahedron, essential for stabilizing the protein. The mutations disrupt the aromatic tetrahedron, leading to misfolding of pVHL. Using biophysical methods we confirmed the *in silico* predictions, demonstrating that mutant pVHL proteins have lower stability than the WT, distort the core domain and as a result reduce the ability of the protein to bind its target HIF-1α. Using bacterial pVHL-EGFP based assay we screened for osmolytes capable of restoring folding of mutant pVHL. Among them, Arginine was the most effective and was verified by *in vitro* assays as a potent re-folder of pVHL. This resulted in functional restoration of the mutant proteins to the level of the WT.

## Introduction

Tumorigenic mutations in the von Hippel-Lindau (*VHL*) gene are associated with the VHL syndrome, an autosomal dominant disorder, which increases susceptibility to various tumors, both benign and malignant, including central nervous system haemangioblastomas, renal cysts and renal cell carcinoma (RCC) and phaeochromocytoma [1], [2], [3]. Notably, *VHL* is inactivated in approximately 80% of sporadic RCC, the most common form of kidney cancer [4], [5]. The VHL tumor suppressor protein is the substrate recognition subunit of a complex comprising pVHL as well as Elongin C and B (VCB) [6], [7], [8]. This complex functions as part of an SCF-like ubiquitin-ligase that promotes the destruction of target proteins required for growth and vascularization of solid tumors [9], [10], [11]. The best known substrate of the VCB complex is Hif-1α, which is involved in cell response to oxygen levels [12], [13], [14]. Upon decrease in oxygen levels residues P564 and/or P402 in Hif-1α are hydroxylated and interact with Y98 in pVHL leading to VCB mediated E3 degradation of Hif-1α [15], [16], [17]. pVHL has a number of HIF-independent functions as well. For example, pVHL interacts with MDM2 and suppresses its ability to ubiqutinate p53, resulting in p53 accumulation and consequent apoptosis [18]; it can also act as an adaptor to bind CK2, which inactivates the NF-κB agonist CARD9, leading to inhibition of NF-κB signaling and overall inhibition of cell survival [19]. pVHL interacts also with collagen IV (Col IV), Kinesin 2 and fibronectin to ensure proper extra cellular matrix (ECM) deposition [20], [21]. pVHL also down-regulates atypical protein kinase C (aPKC), which secondarily results in decreased levels of JUNB (an antagonist of JUN), thus permitting JUN-dependent neuronal apoptosis [22].

Over 800 different mutations have been identified in the *VHL* gene most of them are missense mutations [23]. Analysis of some of them enabled identification of regions of pVHL important for its function. Thus, mutations in its BC box affect its association with Elongin B and C [11]. The solved crystal structure of VCB [24] further identified a putative ubiquitin ligase interaction site on a hydrophobic surface patch of pVHL, frequently mutated in VHL syndrome patients [23], [25]. Mutations in the Hif-1α interaction site, Y98 and Y112, were shown to affect the thermodynamic stability and Hif-1α binding of the entire VCB complex [26]. Importantly, many tumorigenic mutations in *VHL* lie outside of these defined protein interaction sites, raising the possibility that loss of pVHL function may arise through other mechanisms yet to be identified.

The translation of the *VHL* gene from either of two alternative initiation sites, results in a full-length 30 kDa protein and a shorter 19 kDa form [27], [28], [29], [30]. No crystal structure of unbound pVHL is currently available probably due to its molten globule conformation [31]. Therefore, most of the structural data regarding 19 kDa pVHL has been derived from x-ray crystallography when it is associated with Elongin B and C [24]. This, along with another elucidated structure of the VCB complex, suggest that pVHL consist of two domains, α and β [24], [32]. We speculate that oncogenic point mutations, which are common in VHL patients, reduce the stability of pVHL, leading to disruption of its structure and function. Their analysis can provide valuable insights into the pathogenesis of the disease.

Here, using a computational approach, we identified an aromatic tetrahedron in the core of pVHL, comprising residues that are likely to be crucial for proper structure of the protein. This allowed us to predict how oncogenic mutations in these residues cause misfolding of the protein and disruption of its function. We verified these predictions by *in vitro* structural and functional approaches. Further, we tested whether osmolytes can correct misfolding of mutant pVHL. Osmolytes are small organic molecules, including certain amino acids, polyols, and methylamines, used by various organisms to prevent protein misfolding under stress [33]. Arginine, a known osmolyte, restored folding, structure and function of mutant pVHL *in vitro*.

## Results

### Modeling of Wild Type and Mutant pVHL Reveals an "Aromatic Tetrahedron" in its Core

**Figure 1:**
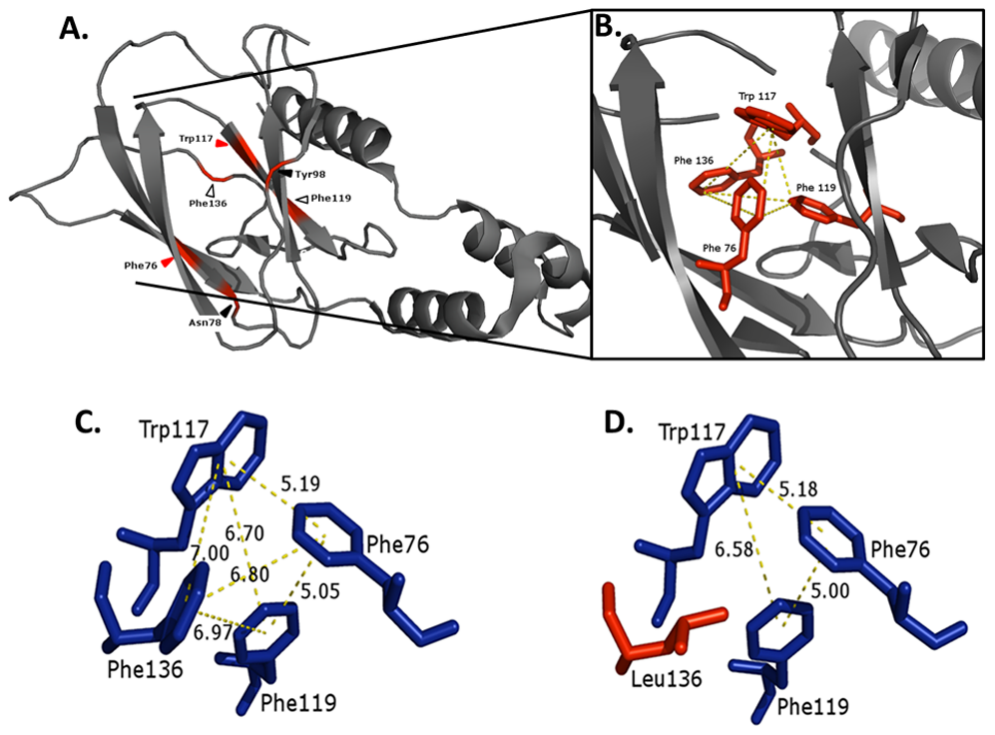
The “aromatic tetrahedron” in the core of pVHL. (**A**) The α and β domains of pVHL. Red arrowheads indicate residues comprising the aromatic tetrahedron. Black arrowheads indicate residues mutated in study. Blank arrowheads indicate residues comprising the aromatic tetrahedron that were also mutated. (**B**) Zoom in on the core of the β domain showing the aromatic tetrahedron comprised of P76, Y117, F119, F136 (red) that map to the S1, S5 and S6 β strands, respectively (**A**). The impact on the aromatic tetrahedron of significant missense mutation shown by the aromatic interaction distances (yellow dashed line). (**C**) WT; (**D**) F136L.

The crystal structure of human pVHL complexed with Elongin C and B (PDB code 1LM8) [32] was chosen for *in silico* evaluation of structural aspects of the wild type pVHL. pVHL comprises a hydrophobic β domain and a hydrophilic α domain. The α domain, which has been the primary focus of structural studies of pVHL, consists of three α helices. Together with an additional helix from Elongin C they make up part of the VCB E3 ubiquitin ligase complex. The β-domain of pVHL consists of a seven-stranded β sandwich, S1–7 (residues 63 to 154) and an α-helix, H4 (residues 193 to 204) that packs against one of the β sheets through hydrophobic interactions. The core of the β domain is stabilized due mainly to hydrophobic and aromatic interactions which play a major role in the core-packing of folded proteins [34] (Figure 1A,B). We identified an “aromatic tetrahedron” comprised of F76, W117, F119, and F136, which map to the S1, S5 and S6 β strands, respectively. These strands link all of the β strands of the β domain to each other, thus greatly contributing to the structural integrity of this domain as well as of the protein as a whole (Figure 1A,B). The distances between the amino acids, which make up the aromatic tetrahedron are in the range of 5.05–7.00 Å. This range is in agreement with the predicted distances at which aromatic-aromatic interactions occur, 4.5–7 Å [34]. We suggest that these aromatic-aromatic interactions are the key forces preserving the β sandwich and the overall structure of pVHL.

We chose four common oncogenic missense mutations in the *VHL* gene and examined their effect on the core packing of pVHL (Figure 1A). Two of them are part of the aromatic tetrahedron (F119L and F136L), one is located on one of the loops far from the aromatic tetrahedron (N78S) and one is the amino acid that directly binds the hydroxyproline in HIF-1α (Y98H). Structure prediction of four homolog models of pVHL, with N78S, Y98H, F119L and F136L oncogenic mutations was conducted (see Figures S1 and Tables S1, S2). The N78S and Y98H mutant protein had virtually identical structure to the WT protein, while the other two, F119L and F136L, showed major changes of the aromatic tetrahedron, i.e. loss of ∼50% the aromatic interactions (Figure 1C,D). We next characterized the WT and these oncogenic mutants by *in vitro* studies.

### Structural and Functional Effects of Oncogenic Mutations on the pVHL Protein

#### The effect on pVHL folding

**Figure 2:**
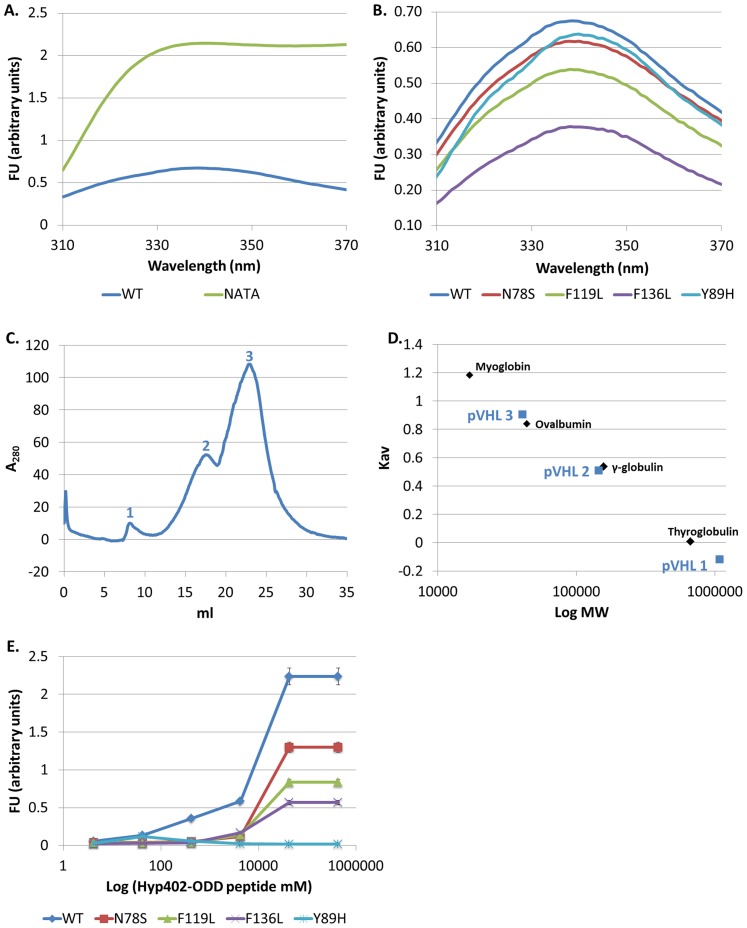
Structure and function of pVHL harboring oncogenic mutations. **(A), (B) Intrinsic fluorescence assay.** Fluorescence emission spectra measured at 25°C. Protein concentration was 5 µM. The excitation wavelength was 295 nm. (**A**) WT in high salt buffer (blue). NATA (green) concentration was adjusted to the concentration of tryptophans in the protein. (**B**) Intrinsic fluorescence of WT and mutant pVHL in high salt buffer. WT (blue), N78S (red), F119L (green), F136L (purple) and Y98H (turquoise). (**C**)**,** (**D**) **Compactness of the tertiary structure of WT pVHL.** Size exclusion preformed with high salt buffer (**C**) Elution curves of WT pVHL (blue). (**D**) Calibration curves generated for molecular weight standards (Kav versus Log Mw). Apparent molecular mass of WT pVHL (blue) was calculated with the curve equation. Molecular weight standard proteins (black): Myoglobin (17 kDa), Ovalbomin (44 kDa), γ-globulin (158 kDa) and Thyroglobulin (670 kDa). (**E**) **Functional analysis of WT and mutant pVHL.** Binding of WT or mutant pVHL to TAMRA labeled Hyp402-ODD target peptide. Fluorescence of TAMRA at 580 nm was measured as a function of log Hyp402-ODD concentration (mM). pVHL protein concentration was 500 nM; WT (blue), N78S (red), F119L (green), F136L (purple) and Y98H (turquoise).

Misfolding of mutant pVHL play a key role in the molecular etiology of the VHL syndrome [35]. The effect of oncogenic mutations on the folding and thermodynamic stability of pVHL itself, unbound to Elongin B and C, has not been studied to date. For that purpose we capitalized on the two tryptophan residues in pVHL, both of which are located in the core of the β domain of the protein. We assessed their relative exposure to the solvent by comparing the intrinsic fluorescence of WT and mutant pVHL to that of N-acetyltryptophanamide (NATA) at a comparable molar concentration of the tryptophan fluorophores (Figure 2A). Fluorescence of NATA provides a reference for the maximal tryptophan fluorescence [36], [37]. The intensity of the fluorescence spectrum of WT pVHL in high salt buffer was substantially lower compared to that of NATA, suggesting considerable quenching of the two tryptophans in the native environment of pVHL.

Circular dichroism (CD) studies provided additional insight into the structure of WT pVHL. Far-UV CD spectra of the WT protein in high salt buffer (Figure S2A) reveals spectral signals consistent with a protein having an intact secondary structure, comprising both α-helices and β-sheets. This is consistent with the secondary structure of pVHL expressed in the presence of Elongin B and C [31]. Furthermore, the study of one of the pVHL mutants (F136L) reveals spectral signals consistent with a highly disordered protein. The near-UV CD spectra shown in Figure S2B reveal no significant signals originating from aromatic side chains of the WT or mutant F136L proteins. This suggests a lack of tertiary structure of the soluble, unbound protein.

The oncogenic mutants examined displayed lower intrinsic fluorescence than the WT. At 25°C the N78S mutant protein showed a small reduction (0.85 fold) in fluorescence intensity as compared to the WT protein (Figure 2B). In contrast, the F119L and F136L mutant proteins displayed a larger decrease (1.5 and 2 fold, respectively) in fluorescence intensity compared to the WT (Figure 2B). This lower fluorescence intensity reflects exposure of the tryptophan residues in the mutant proteins to the aqueous environment as opposed to their buried position in the hydrophobic interior of the WT protein, indicating misfolding of the mutants. For the Y98H mutant small reduction (0.85 fold) and red shift in fluorescence intensity were observed very similar to those recorded for the N78S mutant (Figure 2B).

Propensity for denaturation, either thermal or chemical using urea, was used to evaluate the structural stability of the proteins. Thermal melting point (T_m_°C) was calculated for the WT and mutant pVHL proteins (Table 1). The T_m_ for WT pVHL was very low, 27°C. The mutant proteins N78S and Y98H had lower T_m_ and that of the mutants F119L and F136L was further reduced (16 and 6°C, respectively, Table 1). Intrinsic fluorescence of the WT protein was next examined as a function of the concentration of a chemical denaturant, urea. ∼1 M urea was sufficient for unfolding 50% of the WT protein at 20°C. This agrees with our thermal denaturation analysis revealing a very unstable WT protein. The mutants F119L and F136L are misfolded by the chemical denaturation (Table 1). Taken together, these results confirm that F119L and F136L are misfolded proteins.

**Table 1:** Equilibrium denaturation of WT and mutant pVHL.

With Arginine		Without Arginine		
[U]^50%^	T_m_ (°C)	[U]^50%^	T_m_ (°C)	
1.44	35.97	1.37	27.10	**WT**
1.31	34.44	1.43	22.34	**N78S**
0.70	32.83	0	16.18	**F119L**
1.05	33.92	0	6.11	**F136L**
1.24	33.62	1.30	20.71	**Y89H**

Data are from normalized fluorescence emission at 337 nm after excitation in 295 nm. Data were collected in high salt buffer in the presence and absence of Arginine.

Protein concentration was 5 µM.

**T_m_-** Melting temperature was calculated from intrinsic tryptophan fluorescence (337 nm) as a function temperature (4–80°C).

**[U]^50%^-** Chemical denaturation by urea was calculated from intrinsic tryptophan fluorescence as a function Urea concentration (0–8 M) at 20°C**.**

#### Misfolding of pVHL leads to its aggregation

We used size exclusion chromatography (SEC) to estimate the hydrodynamic dimensions of the WT and mutant pVHL proteins. It also allowed us to elucidate the compactness of the their tertiary structure [38]. SEC was performed in high salt buffer. The WT pVHL protein eluted at three individual peaks (volumes 8.06, 17.55 and 23.55 ml, Figure 2C), which represent three different protein species: large aggregates (>1000 kDa, above void volume), small aggregates (145.41 kDa) and monomers (40.8 kDa) (Figure 2C,D and Table 2). The calculated Stokes radius (Rs) for WT pVHL is 2.9 nm (calculated with curve's equation) (Table 2). These values agree with previously reported data, which suggested that pVHL is a molten globule protein [31], and are larger than the theoretical values of WT pVHL 19 isoform (19 kDa, 1.9 nm).

**Table 2:** Hydrodynamic dimensions of WT and mutant pVHL proteins.

With Arginine		Without Arginine				
Rs (nm)	Molecular weight (kDa)	Rs (nm)	Molecular weight (kDa)			
–	–	**3**	**3**	**2**	**1**	**Peak no.**
1.64	22.81	2.94	40.83	145.41	>1000	**WT**
1.66	24.46	3.12	46.07	142.67	>1000	**N78S**
1.46	22.48	3.22	49.51	171.16	>1000	**F119L**
1.46	23.25	3.31	52.87	158.60	>1000	**F136L**
1.52	23.80	3.27	51.05	154.95	0	**Y89H**

Size exclusion was performed in high salt buffer in the presence and absence of Arginine. Apparent molecular mass of WT and mutant pVHL with (left) and without Arginine (right) was calculated with the curve equation Kav *versus* Log Mw. Apparent Rs of WT and mutant pVHL with (left) and without Arginine (right) was calculated with the curve equation √-logKav versus Rs(nm).

The mutant pVHL proteins eluted faster than the WT (Table 2). Each of the mutants N78S, F119L and F136L eluted as three protein species as observed for the WT pVHL (Table 2). In contrast, the Y98H mutant eluted only as two protein species: a small form of soluble aggregates and monomers (Table 2).

Differences were observed also between the molecular weight of the monomers of the WT and all mutant proteins. The molecular weight of the mutants was larger than that of the WT (46–52 versus 40 kDa, respectively, Table 2). Similarly, the radii of the monomers were larger for these mutants than for the WT (3.1–3.3 vs. 2.9 nm, respectively, Table 2). These results agree with our intrinsic fluorescence results and indicate that the corresponding oncogenic mutations cause protein misfolding and affect the compactness of the protein tertiary structure.

#### Loss of pVHL function

pVHL targets HIF-1*α* for proteasomal degradation under normal oxygen level (normoxia). The ODD domain of human HIF-1*α*, located at residues 401–603, is required for this degradation. This region overlaps two VHL-binding sites and contains two essential proline residues (P402 and P564) that are hydroxylated by prolyl hydroxylase (PHD) under normoxic conditions [39], [40], [41].

For assessing whether the mutant pVHL proteins are less active, we designed an ELISA-based binding assay. For this purpose we synthesized a rhodamine-labeled peptide, termed Hyp402-ODD, comprising the amino acid sequences surrounding P402 in HIF-1*α*, which carried a hydroxylated P402. Increasing concentrations of Hyp402-ODD peptide were incubated, with either WT or mutant pVHL proteins, or with BSA as a negative control, and fluorescence intensity was measured.

The results indicate that, as expected, WT pVHL binds the Hyp402-ODD peptide at high affinity whereas the Y98H mutant protein does not bind it (Figure 2E). The pVHL mutants N78S, F119L and F136L bound the Hyp402-ODD at relatively lower affinity (2, 2.5, and 5 fold, respectively, less than the WT, Figure 2E). These results indicate that although these three mutants carry the natural residue (Y98) required for binding the Hyp402-ODD target peptide, the structural rearrangements caused by the corresponding mutation in each of them disrupts the binding affinity of pVHL to its target. Thus, these oncogenic mutations at positions which are not directly involved in HIF-1*α* binding (as Y98H) cause a significant reduction in pVHL function due to disruption of its structure.

### A Bacterial Assay Indicates that Arginine is a Potent Refolder of pVHL

**Figure 3:**
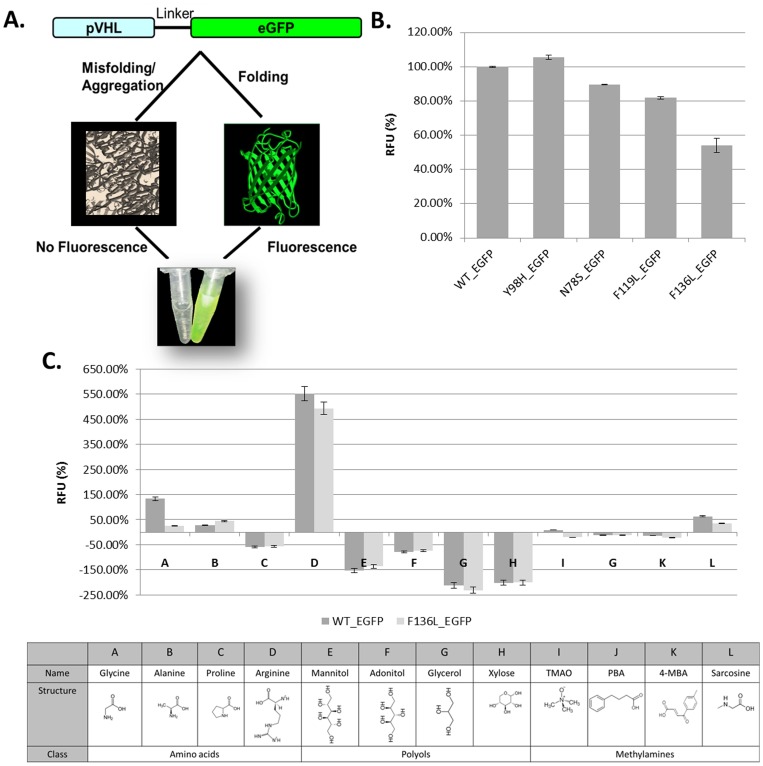
pVHL folding in bacteria. (**A**) Schematic depiction of the properties of pVHL-EGFP fusion proteins. Insoluble protein forms aggregates (left) and prevents the EGFP portion of the fusion protein from forming its native fluorescent structure. However, well folded, soluble pVHL, folds normally and does not aggregate, which enables EGFP to form its native green fluorescent structure (right). (**B**) Fluorescence spectra of *E. coli* cells expressing the pVHL-EGFP fusion WT and mutants. Absolute fluorescence intensities at 520 nm are shown normalized compared to WT pVHL in LB. (**C**) Screening results of several selected chemical chaperon candidates, from three classes. Fluorescence spectra of *E. coli* cells expressing the pVHL-EGFP fusion WT (gray) and F136L mutant protein (light gray) in the presence of chemical compounds. Absolute fluorescence intensities at 520 nm are shown normalized compared to proteins in LB. The classes to which the chemical chaperons belong are shown at the table below.

A bacterial assay has been reported which allows monitoring protein misfolding and aggregation by fusing the protein of interest to green fluorescent protein [42]. When the target protein, is unfolded, it interferes with the early stages of EGFP folding into its native fluorescent form (Figure 3A). The intensity of the fluorescence signal is proportional to the amount of folded target protein [43]. We over expressed EGFP fused to WT or oncogenic mutant versions of pVHL in *E. coli* and measured EGFP signal. Fluorescence of all mutants, except Y98H, was found to be lower than that of WT pVHL, indicating that they are less stable than the WT in bacteria (Figure 3B).

Osmolytes were reported to aid in refolding proteins and decreasing their aggregation [44]. Using the pVHL-EGFP system we screened for osmolytes that would increase folding of mutant pVHL. Molecules belonging to three classes of osmolytes: amino acids, polyols and methylamines were tested. Four molecules were tested from each class. We began by examining WT pVHL and the F136L mutant version. As can be seen in Figure 3C, polyols (such as manitol, adonitol, glycerol and xylose) lowered EGFP intensity, presumably reflecting increase in misfolding and aggregation of the WT and F136L pVHL proteins. Methylamines (e.g. TMAO, PBA and 4-MBA) had no effect on EGFP signal, except for sarcosine, which enhanced EGFP fluorescence, suggesting restoration of folding and inhibition of aggregation of both proteins examined. Among the amino acids tested we found that proline lowered whereas glycine and alanine moderately enhanced EGFP fluorescence. Importantly, Arginine caused the highest EGFP fluorescence, six fold more than glycine and alanine (Figure 3C).

### Effect of Arginine on pVHL Harboring Oncogenic Mutations

**Figure 4:**
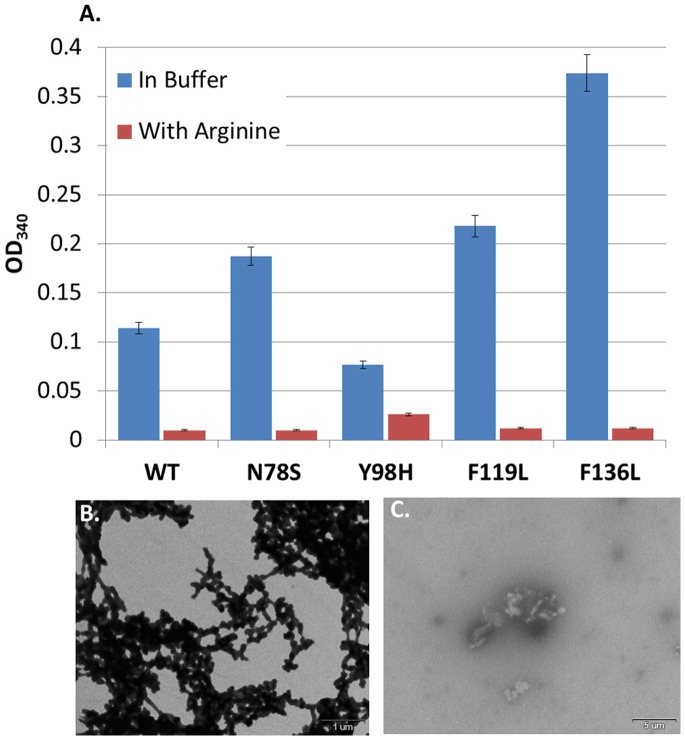
Refolding of WT and mutant pVHL by Arginine. (**A**) Refolding of WT and mutant pVHL in the presence of Arginine. Protein aggregation was monitored by the absolute absorbance at 340 nm in high salt buffer lacking Arginine (blue) or supplemented with Arginine (red). Protein concentration - 5 µM; Arginine concentration 0.6 M. (**B**)**,** (**C**) Morphology of WT pVHL after refolding in the present of high salt buffer without (**B**) or with Arginine (**C**). Transmission electron micrographs shown are representative of multiple fields recorded for each of several grids prepared in at least three independent experiments.

Since misfolding leads towards aggregation we employed a protein refolding assay [45] to examine the differences in aggregation propensity between the WT and mutant pVHL variants in the presence or absence of Arginine. The degree of aggregation was monitored by the absorbance of the sample at 340 nm. In the presence of buffer we observed aggregates of both the WT and all mutant pVHL proteins examined (Figure 4A,B). The mutants N78S, F119L and F136L displayed much greater aggregation propensity than the WT protein (e.g. two fold for N78S and F119L, and four fold for F136L), while Y98H exhibited a 2-fold decrease compared to the WT (Figure 4A). Arginine reduced the aggregation of the WT as well as all mutant pVHL proteins examined as much as tenfold (Figure 4A). To verify that the species generated in solution were indeed aggregates we examined their morphology using transmission electron microscopy (TEM). The WT protein refolded in buffer exhibited amorphic thick aggregates (Figure 4B), whereas in the presence of Arginine, no aggregates were observed (Figure 4C). Similar results were obtained for all mutant proteins with and without Arginine.

**Figure 5:**
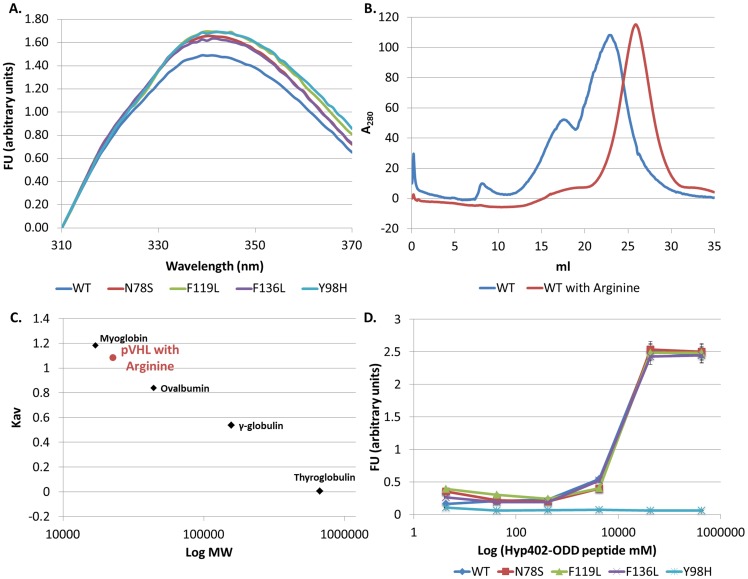
The effect of Arginine on pVHL harboring oncogenic mutations. **(A) Intrinsic fluorescence in the presence of Arginine.** Fluorescence emission spectra measured at 25°C. Protein concentration was 5 µM. The excitation wavelength was 295 nm. Intrinsic fluorescence of WT and mutant pVHL in the presence Arginine. WT (blue), N78S (red), F119L (green), F136L (purple) and Y98H (turquoise). (**B**)**,** (**C**) **Compactness of the tertiary structure of WT pVHL in the presence of Arginine.** Size exclusion preformed with high salt buffer in the presence of Arginine. (**B**) Elution curves of WT pVHL with (red) and without Arginine (blue). (**C**) Calibration curves generated for molecular weight standards (Kav versus Log Mw). Apparent molecular mass of WT pVHL with Arginine (red) was calculated with the curve equation. Molecular weight standard proteins (black): Myoglobin (17 kDa), Ovalbomin (44 kDa), γ-globulin (158 kDa) and Thyroglobulin (670 kDa). (**D**) **Functional analysis of WT and mutant pVHL in the presence of Arginine.** Binding of WT or mutant pVHL to TAMRA labeled Hyp402-ODD target peptide. Fluorescence of TAMRA at 580 nm was measured as a function of log Hyp402-ODD concentration (mM). pVHL protein concentration was 500 nM; WT (blue), N78S (red), F119L (green), F136L (purple) and Y98H (turquoise).

Adding Arginine to the WT protein increased the intrinsic fluorescence intensity by two fold (Figure 5A). The intrinsic fluorescence of all mutant proteins tested increased 3–6 fold in the presence of Arginine (Figure 5A). Interestingly, the compactness attained by the mutants in the presence of Arginine, as evident in their increased fluorescence intensity, was even higher than that of WT pVHL in buffer (Figure 2B) and reached the degree of fluorescence attained by WT pVHL in the presence of Arginine. Adding Arginine increased the T_m_ of all proteins examined, including the WT, to >30°C and also enhanced the resistance of the mutants F119L and F136L to chemical denaturation (Table 1).

SEC was used to estimate the aggregation forms and the hydrodynamic dimensions of these proteins in the presence of Arginine. Adding Arginine to the buffer resulted in a single, significantly higher, elution peak, at 26.3 ml (Figure 5B) for all proteins, mutant and WT. Arginine eliminated all soluble aggregate forms for all proteins examined and they accumulated only at the monomeric form similar to a compactly folded 22.8 kDa WT protein (Figure 5B, C and Table 2). Under these conditions the monomers of all proteins examined attained a lower molecular weight in the presence of Arginine (∼23 kDa) and a smaller radius (∼1.5 nm) (Figure 5B, C and Table 2) compared to these proteins in high salt buffer. Note that as revealed by the intrinsic fluorescence, Arginine caused the mutant proteins to have a conformation more compact even than that of the WT protein in buffer.

Testing the function of the proteins in the presence of Arginine demonstrated dramatic improvement of their ability to bind the Hyp402-ODD target peptide to a level characteristic of WT pVHL, indicating full functional restoration (Figure 5D). As expected, Arginine had no restorative effect on target binding by the Y98H mutant, which affects the residue required for contact with the target.

Taken together, our *in silico*, *in vitro* and bacterial assays corroborate each other and provide multifaceted evidence for structural restoration and functional rectification of the tumorigenic mutant pVHL proteins.

## Discussion

### pVHL is an IDP

The instability of pVHL has been first deduced from its tendency to fold only whilst bound to its partners Elongin B and Elongin C [46]. Our earlier *in vitro* studies have shown that pVHL has a secondary structure composed of α helices as well as β-sheets and a large percentage of random coils (Figure S2, [31]). near-UV CD spectra indicated lack of tertiary structure, which led to the conclusion that pVHL is a molten globule protein (Figure S2, [31]). Yet, direct demonstration that pVHL is functional in its molten global state has not been reported.

In the present study we confirmed that pVHL is a molten globule under native conditions based on a series of biophysical assays including intrinsic fluorescence, thermal and chemical denaturation and SEC. We determined that the WT pVHL protein has low thermodynamic stability (T_m_ of 27°C) and higher molecular weight and hydrodynamic radius than expected, all of which are characteristic of intrinsically disordered proteins (IDPs) [47]. The low thermodynamic stability of pVHL and its molten globule state may account for its tendency to aggregate as observed in cultured cells [48]. Indeed, size exclusion of WT pVHL in solution revealed several distinct peaks which, when resolved on SDS-PAGE, were found to correspond to soluble aggregates in addition to monomers. Furthermore, by demonstrating that WT pVHL binds its Hyp402-ODD target peptide we showed, for the first time, that it is fully functional in its molten globule state, a genuine feature of IDPs. The molten globule nature confers pVHL with structural versatility, characteristic of IDPs [49], that enables it to interact with various proteins for carrying out its multiple cellular functions in addition to targeting HIF-1α for ubiquitin-mediated degradation [50].

### pVHL and Cancer

To date more than 800 cancer-associated mutations have been identified in the *VHL* gene. Most of them are missense mutations located within the pVHL core [51]. Two of them where shown to directly affect the residues through which pVHL binds its partners, namely mutations in R167 and in Y98 which diminished binding to Elongin C and HIF-1α, respectively [11], [16]. The biophysical and thermodynamic effects of other mutations in the core domain have not been studied. We speculated that mutants in residues not involved in binding partner proteins affect the folding of pVHL and as a consequence impair its function. Our work on the four frequent oncogenic mutations, two in the aromatic tetrahedron that we have identified, one out of it, as well as the Y98H supported this hypothesis. The *in silico* predictions, verified by *in vitro* analyses, indicated that all four mutations greatly impair the stability and function of the corresponding mutant pVHL proteins. Y98 contact mutations were shown to affect the stability of the VCB complex as a whole [26]. We found that Y98H affects the structural stability of pVHL also when unbound to its partners in the VCB complex. Interestingly, amorphous aggregation, revealed by size exclusion analysis, was found for all proteins tested but not for the version carrying Y98H. Our work has shown that oncogenic missense mutations that disrupt the aromatic tetrahedron result in loss of folding, stability and function of pVHL. By extrapolation, we propose that most of the 800 missense mutations in the *VHL* gene also lead to misfolding of the protein contributing to the cancerous symptoms of the VHL syndrome.

Various human diseases are attributed to protein misfolding and aggregation. The cancer microenvironment exposes malignant cells to a variety of stressful conditions that may further promote protein misfolding [52]. Disruption of protein function due to misfolding has been demonstrated in tumors associated with missense mutations in various proteins including Src family kinases, p53, mTOR and C-terminus of HSC70 interacting protein (CHIPs) [53]. Approaches aimed at correcting their misfolding may therefore be useful therapeutic strategies.

### Arginine as Folding Inducer of pVHL

Osmolytes have been proposed as means for restoring folding and function of misfolded proteins. In particular, they could serve to correct folding of mutant proteins associated with various diseases [52]. We attempted to apply this approach to the misfolded pVHL mutants described in this work. Among the compounds examined Arginine proved to be the best. Arginine was highly effective in refolding of both the WT and the mutant pVHL proteins in our bacterial system screen and in the refolding assay. By intrinsic fluorescence assays we showed that Arginine rendered the core of these proteins more condensed and their T_m_ was remarkably elevated. It is noteworthy that Arginine stabilized even the WT pVHL as shown by the increase of its T_m_ from 27 to 35°C. The effect of Arginine on the pVHL mutants was very strong bringing all of them close to the T_m_ of the WT. pVHL, not bound to its complex (i.e. VCB complex), was shown to have a molten globule conformation seen in SEC larger than its calculated molecular weight (∼40 kDa vs. ∼19 kDa, respectively). WT pVHL as well as the mutants attained a lower molecular weight in the presence of Arginine (Fig. 4). Thus, Arginine had a strong effect on the molten globule conformation of WT pVHL and pushed it into the native state, i.e. a more compact conformation. Note that this change in conformation did not interfere with the function of binding Hif-1α (Figure 5). The SEC analysis indicated that Arginine also inhibited the formation of pVHL aggregates. This effect of Arginine on the folding of the structural mutant proteins led to restoration of their function in binding the hydroxylated Hyp402-ODD target peptide. The binding ability of the mutant proteins was restored to the level of the WT. In contrast, as expected, no functional restoration was observed for the contact mutant Y98H.

According to “the rapid hydrophobic collapse model” [54], hydrophobic interactions are the driving force for protein folding and aggregation. It postulates that self-interaction of Arginine leads to the formation of clusters which, due to their size, crowd out the protein-protein interactions. In addition, Arginine affects surface tension of the aqueous solution and thus increases the solubility of amino acids (for review see [55]). Furthermore, some specificity of Arginine for side chains of the protein may be responsible for preventing protein aggregation. The guanidinium group is known to interact with the aromatic side chains of proteins [56]. Arginine may interact via this group with aromatic side chains, which are buried in the protein and responsible for the aggregation of unfolded or partially folded protein structures. Thus, the mode of action of Arginine on unfolded or partially folded proteins may be mediated by interaction via its guanidinium group with their aromatic side chains. This would stabilize the structure of these proteins and prevent their aggregation.

### A model for Folding-misfolding Pathway of pVHL

**Figure 6:**
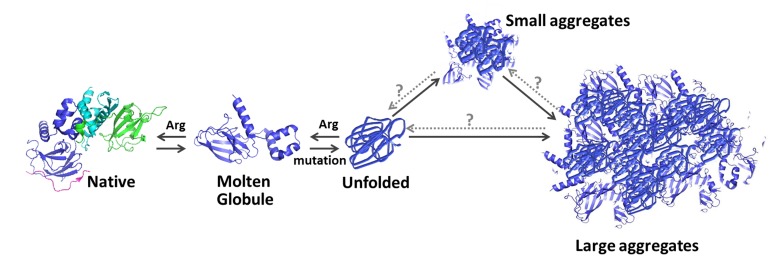
Proposed model of folding/misfolding pathway of pVHL. In the absence of the nanny proteins (VCB complex, green and turquoise) pVHL conformation is molten globule. Then, it can either bind the nanny proteins resulting in a restored native conformation, or it can further unfold. The unfolded pVHL can refold into a molten globule protein; or can form small or large aggregates. Aggregated pVHL is presumably rapidly degraded. Missense mutations in pVHL enhance its tendency to unfold and aggregate. Presumably, disruption of the aggregates can tilt the equilibrium towards the native state.

Our results suggest a model for the dynamics of a folding-misfolding pathway of pVHL (Figure 6) and suggest a novel approach for intervening in it for therapeutic purposes. Maintaining normal folding requires association of the appropriate nanny proteins (e.g. VCB complex) [57]. In their absence, pVHL folds into a molten globule state. In its molten globule conformation pVHL can either bind back the nanny proteins, resulting in a restored native state, or it can further unfold. Unfolded pVHL can refold into a molten globule protein, or can form small or large aggregates as evident by the refolding assays and SEC (Figure 2D, 4A,B and Table 2). Mutations in pVHL enhance its tendency to misfold and aggregate *in vitro*. If this happens in cells molecular chaperons such as TRiC/CCT and Hsp70 can prevent this aggregation [35], [48], [58]. Mutant versions, such as F136L and F119L, which are highly destabilized, produce substantially more aggregates. The observation that mutants with a higher propensity to unfold aggregate more massively suggests that aggregation occurs from the unfolded state and not directly from the molten globule conformation.

Our *in vitro* and bacterial findings indicate that osmolytes, such as Arginine, can revert unfolded and molten globule conformations of pVHL back into the native state, accompanied by restoration of its function of binding Hif-1α. We further speculate that disruption of the pVHL aggregates can tilt the equilibrium towards the native state.

## Experimental Procedures

### *In Silico* Analysis

The VHL protein sequence was retrieved from UniProt and the VHL protein structure was obtained from The Protein Data bank (PDB code: 1LM8) [32], [59]. A protein sequence for each amino acid mutation was generated (N78H, F119L, F136L, Y98H), and the effect of each amino acid mutation on the protein structure of VHL was predicted using I-TASSER [60] (URL: http://zhanglab.ccmb.med.umich.edu/I-TASSER/). The I-TASSER server participated in the Server Section 9th CASPs (2010), and was ranked as the No 1 server [61]. The models produced by I-TASSER were evaluated and visualized using PyMOL (DeLano Scientific; URL: http://www.pymol.org/). PIC, The Protein Interactions Calculator (URL: http://pic.mbu.iisc.ernet.in/) was used to generate residue interaction networks. These networks were useful for evaluation of structural changes induced by amino acid substitutions. Default minimum distances were used to define interaction types: 4.5 Å for π–π interaction; and 6.0 Å for π-cation interaction [34].

### Gene Subcloning and Mutagenesis

The open reading frame (ORF) of pVHL, kindly provided to us by Dr. Nikola Pavletich (Memorial Sloan-Kettering Cancer Center, New York), was cloned into the pET14b vector (from Novagen) using *Nde*I and *Xho*I (TaKaRa) restriction enzymes. Site specific Mutagenesis was performed with inverse PCR using designed primers to replace the native amino acid.

### Protein Expression and Purification

The vector was transformed to Rosetta strain of *E. coli*. Transformed cells were grown at 37°C, 200 rpm, in 2XYT medium (Difco) under antibiotic selection (100 µg/ml ampicillin). The cells were grown to an OD600 = 2.5 and induced by adding 1 mM of Isopropyl β-D-1-thiogalactopyranoside (Sigma-Aldrich) for 3 hours at 37°C. The pVHL protein was found to be insoluble in the bacterial inclusion bodies. The cells were broken using a high-pressure homogenizer and adding 40 mg of lysozyme (Sigma-Aldrich) in TE 50∶20 buffer (50 mM Tris-HCl pH8, 20 mM EDTA). Following disruption, the inclusion bodies were recovered and washed in TE 50∶20 buffer with 1%v/v triton using centrifugation. Inclusion bodies pellet was dissolved in the presence of 6 M guanidine hydrochloride and diluted to 10 mg/ml protein concentration. The protein was refolded from fully reduced and unfolded samples by step-wise dialysis from high denaturant concentration, via middle concentration, and to low concentration of guanidine hydrochloride into high salt buffer (10 mM Tris-HCl pH8.5, 500 mM NaCl). pVHL was purified to >97% purity, and migrated on SDS-PAGE as a 19 kDa protein. Protein concentration was determined from absorbance at 280 nm using ε280 nm = 18,575 cm-1 M-1 for the WT, N78S, F119L, F136L proteins and 17,085 cm-1 M-1 for Y89H protein.

### Protein Refolding

Refolding was initiated by rapid 100-fold dilution of the denatured pVHL WT or mutant protein (5 µM final concentration) into buffer (10 mM Tris-HCl pH8.5) with and without 0.6 M of L-Arginine monohydrochloride (Sigma-Aldrich). The proteins were incubating for 48 h at 7–4°C without any agitation. Aggregation was measured by the turbidity absorbance at 340 nm. Experiments were done in triplicates and error bars are presented.

### Size Exclusion Chromatography (SEC)

SEC experiments were performed using TSKgel-G3000S size exclusion column (TOSOHAAS) with a separation range of 10–1000 kDa connected to an FPLC prime automated liquid chromatography system (Amersham Biosciences). The running buffer used was 10 mM Tris-HCl pH 8, 500 mM NaCl with and without 0.6 M L-arginine monohydrochloride (Sigma-Aldrich). The column was calibrated using gel filtration molecular weight standard (Bio-Rad). The following standards were used for calibration: Thyroglobulin (670 kDa, Rs = 8.6 nm), γ-globulin (158 kDa, Rs = 5.1 nm), Ovalbumin (44 kDa, Rs = 2.8 nm), Myoglobin (17 kDa, Rs = 1.9 nm), Vitamin B12 (1.35 kDa), and Dextran blue (2 MDa).

A 0.5 ml protein sample at a final concentration of 5 µM was filtered and chromatographically analyzed using a flow rate of 0.5 ml/min. Absorbance was monitored at 280 nm, elution volumes were determined from UV chromatogram. The partition coefficient, Kav, was calculated from the elution volume of the sample, Ve, and total bed volume, Vt, using the expression: 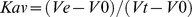. Stokes radius (Rs) for all proteins was calculated by plotting 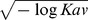 versus known Rs. Calibration curves and equations were established.

### Fluorescence Measurements

Intrinsic fluorescence emission spectra were measured in a Horiba Jobin Yvon FL3-11 spectrofluorimeter (Horiba Jobin Yvon Inc.). Temperature was varied from 4 to 80°C by temperature controller. Excitation was at 295 nm; all slits were set at 5 nm. Spectra were recorded at 1 nm intervals from 310 to 380 nm with a 1s averaging time. Samples in a 1-cm-square cuvette contained 5 µM protein in high salt buffer with and without 0.6 M L-Arginine. All measurements were made using 1-cm square cuvettes and the background fluorescence of buffers alone or buffers supplemented with L-Arginine was subtracted from sample spectra. The bandwidth was 1 nm, and each spectrum shown is the result of three spectra accumulated and averaged.

### Thermal Denaturation

Thermal denaturation studies were carried out using intrinsic fluorescence emission spectra. A solution of 5 µM protein in high salt buffer with and without 0.6 M L-Arginine was heated stepwise at 5°C increments from 4°C to 80°C. Changes in intrinsic fluorescence were scanned from 310 to 380 nm for each step of temperature. Spectra were recorded at 1 nm with a 1s averaging time. All measurements were made using 1-cm square cuvettes and the background fluorescence of buffers alone or buffers supplemented with L-Arginine was subtracted from sample spectra. The bandwidth was 1 nm, and each spectrum shown is the result of three spectra accumulated and averaged.

### Chemical Denaturation

Urea denaturation studies were performed using intrinsic fluorescence emission spectra. A solution of 5 µM protein in high salt buffer with and without 0.6 M L-Arginine was mixed with appropriate amounts of the same solution containing 10 M urea to achieve the appropriate concentration of protein and denaturant. Changes in intrinsic fluorescence were scanned from 310 to 380 nm for each step of urea addition; Spectra were recorded at 1 nm with a 1s averaging time. All measurements were made using 1-cm square cuvettes at 20°C, and the background fluorescence of buffers alone or buffers supplemented with L-Arginine was subtracted from sample spectra.

### Data Analysis

The protein unfolding curves were analyzed using a two-state mechanism. First, unfolding curves for the N↔U transition were normalized to the apparent fraction of the unfolded form, F_U_, using the following equation [62],



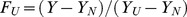
(1)Where Y is the observed variable parameter, and Y_N_ and Y_U_ are the corresponding values for the native and fully unfolded conformations, respectively. The difference in free energy between the folded and the unfolded states, ΔG, was calculated by the following equation,



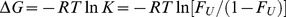
(2)Where K is the equilibrium constant, R is the gas constant, and T is the absolute temperature. The data were analyzed assuming the free energy of unfolding or refolding, ΔG, to be linearly dependent on the urea concentration [63].

### pVHL Function

An ELISA-based binding assay was used. The Hyp402-ODD target peptide was synthesized by Hy Laboratory Ltd (Israel). This peptide (LDLEALA**Hyp**YIPADDDFQLRS) comprised the amino acid sequence around Pro402 in human HIF-1*α* and had this residue hydroxylated as in HIF-1*α*. The peptide was tagged with the fluorophore rhodamine (TAMRA). pVHL protein variants tested were diluted in high salt buffer with and without 0.6 M L-Arginine to 10 µg/ml. 100 µl of the tested pVHL protein was add to each well of an ELISA plate (Costar EIA). Experiments were done in triplicates and error bars are presented. Plates were incubated overnight at 4°C. Wells were washed four times with high salt buffer. Blocking buffer (high salt buffer containing 5%w/v BSA) was added to all wells to block protein binding sites left open in the wells. Labeled Hyp402-ODD target peptide solutions, at increasing concentrations, were added to the wells. Plates were incubated for 1 h at 25°C and were washed thereafter four times with high salt buffer. Fluorescence intensity at λ_580 nm_ was measured following excitation at λ_540 nm_. BSA protein (Amresco) served as a negative control.

### Monitoring Aggregation Using Conjugated Fluorescent Proteins

Cloning the EGFP folding reporter: The ORF encoding wild type 19 kDa pVHL and its mutants were amplified by PCR from the plasmid mentioned above containing wild-type pVHL or its mutants. The amplification products contained the appropriate pVHL constructs with a C-terminal amino acid linker GTGS(GGGS)2GGGAM, removing the stop codon and leaving at its 5' and 3' ends the recognition sequence for *Nde*I and *Nco*I, respectively. The resulting gene fragments were digested with *Nde*I/*Nco*I, and ligated into an *Nde*I/*Nco*I digested pET24b (+) vector containing the EGFP gene downstream. Fluorescence measurements and determination of folding state: The vector expressing either of the fusion proteins was transformed into the *E. coli* strain BL21 and cultures were grown at 37°C in LB medium (Difco) supplemented with 30 mg/ml of kanamycin. At A600 = ∼0.6, the cells were induced with isopropyl-β,D thiogalactopyranoside (IPTG-Sigma Aldrich) to a final concentration of 1 mM, and growth was continued at 16°C for 48 h in the presence or absence chemical candidates. Fluorescence was measured using a 96-well plate reader (Biotek Synergy) (excitation 485/20 nm; emission 528/20 nm) and 96-well plates (COSTAR®3595, Corning Incorporated USA). The cell density was normalized to A600 = 1. The A600 and EGFP normalized relative fluorescence values to the wt protein (% RFU) for each pVHL-EGFP fusion protein were calculated using equation [3], where RFU_(fusion-protein)_, RFU_(background)_ and RFU_(EGFP)_ represent the fluorescence value for the cells expressing pVHL-EGFP fusion protein, the cells expressing pVHL alone and the cells expressing EGFP, respectively:



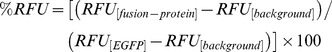
(3)


All experiments were done in triplicates, error bars are presented. Chemical candidates were added when cells were at A600 = ∼0.6 to a final concentration of 150 mM.

## Supporting Information

Figure S1 **(A–E) Effect of missense mutations on the structure of the aromatic tetrahedron** **in pVHL.** The aromatic tetrahedron in WT and mutant pVHL proteins (based on PDB code:1lm8), comprising F76, W117, F119 and F136. Mutated residues are highlighted in red. The impact on the aromatic tetrahedron of each missense mutation shown by the aromatic interaction distances (yellow dashed line). **A.** WT; **B.** F119L; **C.** F136L; **D.** Y98H; **E.** N78S; **(F) Position of the missense pVHL mutations studied.** Crystal structure of pVHL (gray)(PDB ID code 1lm8) showing the N78, Y98, F119 and F136 residues (red). Missense mutations in these residues cause cancer. **(G) Exposure of hydrophobic residues in the missense mutant proteins.** Superimposition of the mutant pVHL structures on the structure of the WT pVHL (blue) (PDB code 1LM8) shown as spheres. Note the overall change in pVHL structure and the resultant exposure of hydrophobic residues (green).
(TIF)

Figure S2 **Far-UV (A) and near-UV (B) CD spectra of pVHL, WT (blue) and mutant F136L (red).** CD spectra measurements were conducted at 25°C. Protein samples were at final concentration of 3 µM pVHL in 10 mM Tris-HCl (pH 8) and 500 mM NaCl.
(TIF)

Table S1 **Output of the Protein Interactions Calculator (PIC).** This table summarizes the PIC output on hydrophobic interactions within 5 angstroms and aromatic-aromatic interactions within 4.5–7 angstroms, relevant to the aromatic tetrahedron.
(DOCX)

Table S2 **RMS deviation of C-α in the 4 models of mutants, as compared to the WT structure.**
(DOCX)

Text S1 **In silico prediction of the effect of oncogenic missense mutations on the structure of pVHL.**
(DOCX)
