# Supplementary figures and images for: Structural Insights into the Folding Defects of Oncogenic pVHL Lead to Correction of Its Function In Vitro
Source: PLoS One. 2013 Jun 20;8(6):e66333. doi: 10.1371/journal.pone.0066333 (PMC3688787; doi:10.1371/journal.pone.0066333)

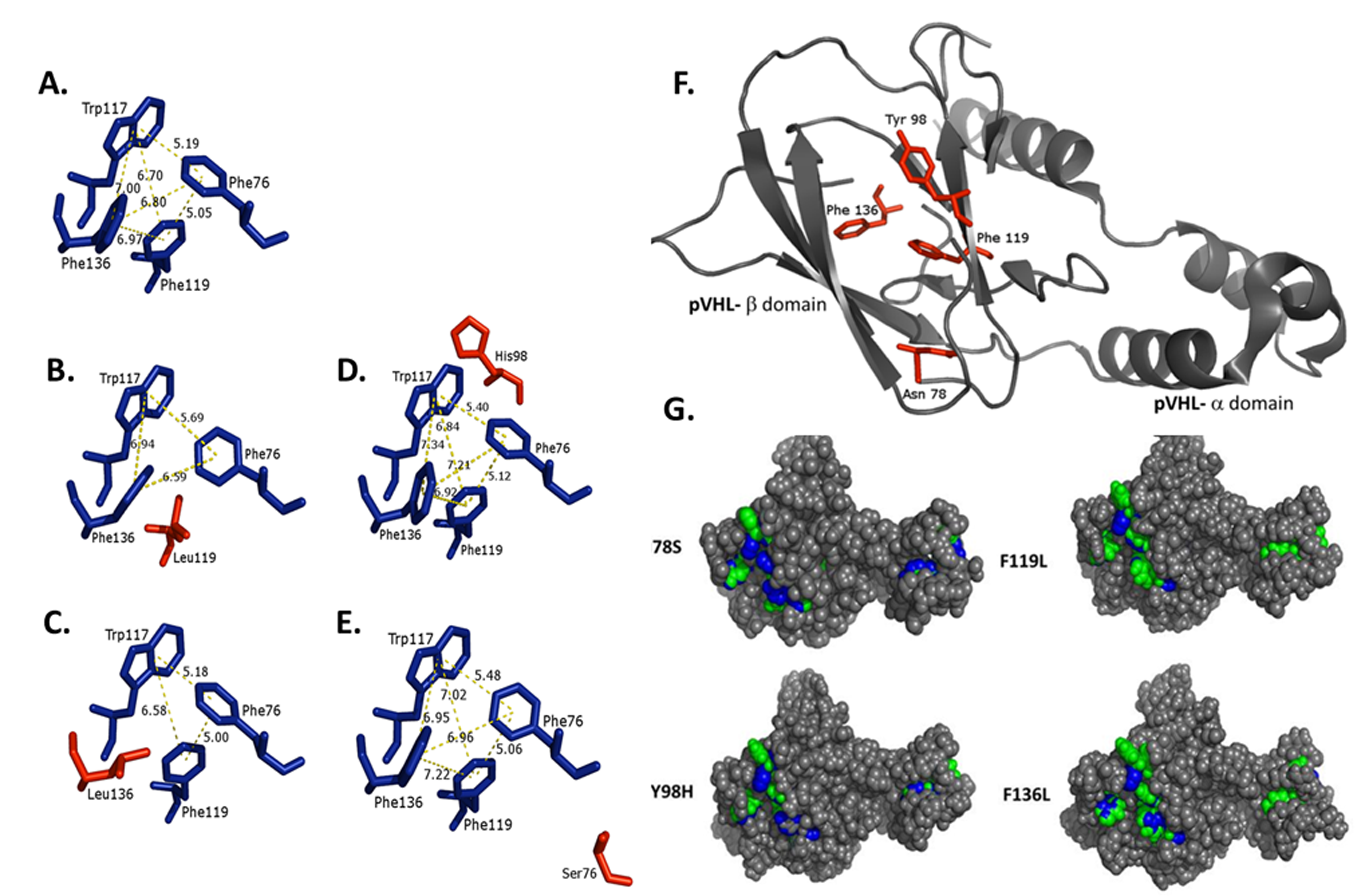

Supplement: Figure S1 — (A–E) Effect of missense mutations on the structure of the aromatic tetrahedron in pVHL. The aromatic tetrahedron in WT and mutant pVHL proteins (based on PDB code:1lm8), comprising F76, W117, F119 and F136. Mutated residues are highlighted in red. The impact on the aromatic tetrahedron of each missense mutation shown by the aromatic interaction distances (yellow dashed line). A. WT; B. F119L; C. F136L; D. Y98H; E. N78S; (F) Position of the missense pVHL mutations studied. Crystal structure of pVHL (gray)(PDB ID code 1lm8) showing the N78, Y98, F119 and F136 residues (red). Missense mutations in these residues cause cancer. (G) Exposure of hydrophobic residues in the missense mutant proteins. Superimposition of the mutant pVHL structures on the structure of the WT pVHL (blue) (PDB code 1LM8) shown as spheres. Note the overall change in pVHL structure and the resultant exposure of hydrophobic residues (green). (TIF) [file pone.0066333.s001.tif]

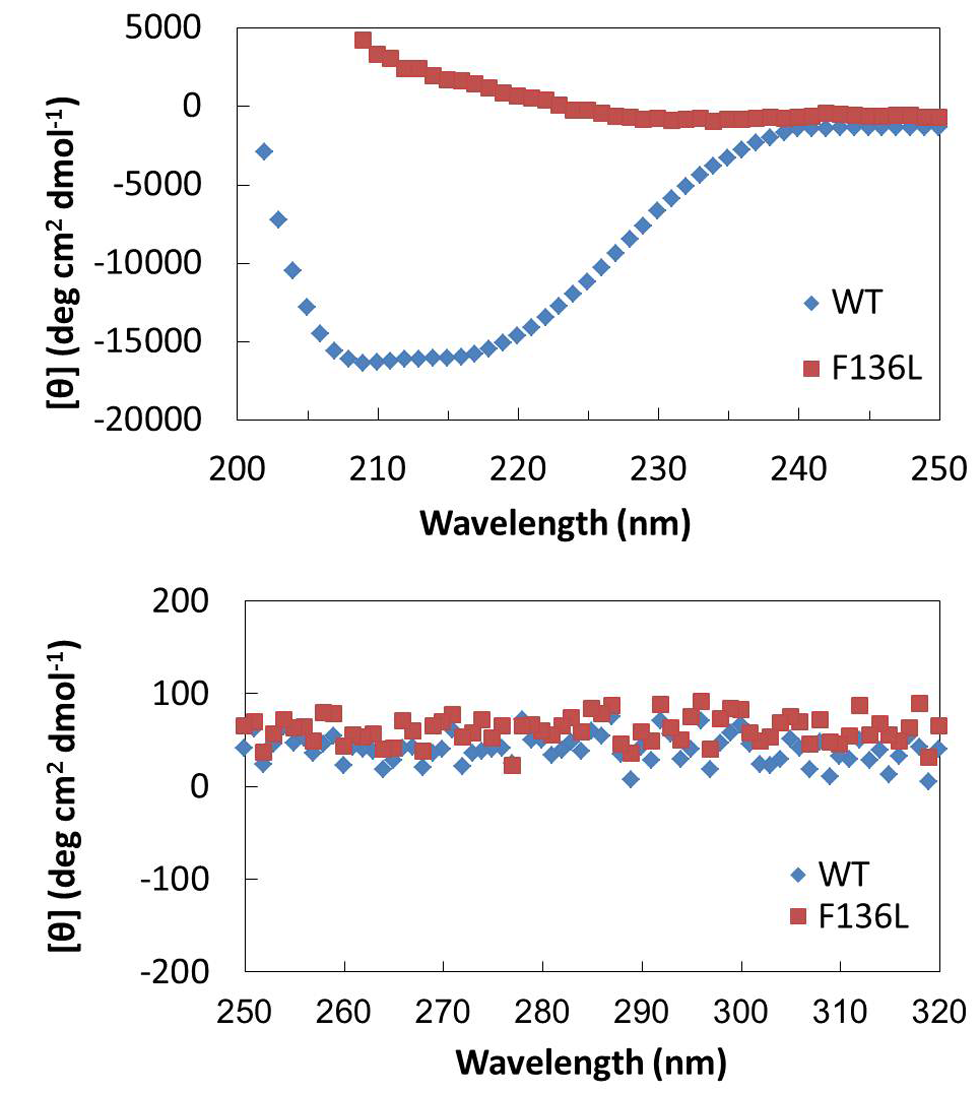

Supplement: Figure S2 — Far-UV (A) and near-UV (B) CD spectra of pVHL, WT (blue) and mutant F136L (red). CD spectra measurements were conducted at 25°C. Protein samples were at final concentration of 3 µM pVHL in 10 mM Tris-HCl (pH 8) and 500 mM NaCl. (TIF) [file pone.0066333.s002.tif]
