# Supplementary material for: Structural Insights into the Folding Defects of Oncogenic pVHL Lead to Correction of Its Function In Vitro
Source: PLoS One. 2013 Jun 20;8(6):e66333. doi: 10.1371/journal.pone.0066333 (PMC3688787; doi:10.1371/journal.pone.0066333)
Supplement: Table S1 — Output of the Protein Interactions Calculator (PIC). This table summarizes the PIC output on hydrophobic interactions within 5 angstroms and aromatic-aromatic interactions within 4.5–7 angstroms, relevant to the aromatic tetrahedron. (DOCX) [file pone.0066333.s003.docx]

**Table S1. Output of the Protein Interactions Calculator (PIC).**

| Hydrophobic Interactions within 5 Angstroms | | | | | Aromatic-Aromatic Interactions within 4.5 and 7 Angstroms | | | | |
| --- | --- | --- | --- | --- | --- | --- | --- | --- | --- |
| pVHL type | **Posi-**  **tion** | **Residue** | **Posi-**  **tion** | **Residue** | **pVHL type** | **Posi-**  **tion** | **Residue** | **Posi-**  **tion** | **Residue** |
| WT | 76 | PHE | 117 | TRP | **WT** | 76 | PHE | 117 | TRP |
| WT | 76 | PHE | 119 | PHE | **WT** | 76 | PHE | 119 | PHE |
| WT | 76 | PHE | 136 | PHE | **WT** | 76 | PHE | 136 | PHE |
| WT | 117 | TRP | 119 | PHE | **WT** | 119 | PHE | 136 | PHE |
| WT | 117 | TRP | 136 | PHE | **WT** | 117 | TRP | 119 | PHE |
| WT | 119 | PHE | 136 | PHE | **WT** | 119 | PHE | 136 | PHE |
| F136L | 76 | PHE | 117 | TRP | **F136L** | 76 | PHE | 117 | TRP |
| F136L | 76 | PHE | 119 | PHE | **F136L** | 76 | PHE | 119 | PHE |
| F136L | 76 | PHE | 136 | LEU | **F136L** | 117 | TRP | 119 | PHE |
| F136L | 117 | TRP | 119 | PHE |  |  |  |  |  |
| F136L | 117 | TRP | 136 | LEU |  |  |  |  |  |
| F136L | 119 | PHE | 136 | LEU |  |  |  |  |  |
| F119L | 76 | PHE | 117 | TRP | **F119L** | 76 | PHE | 117 | TRP |
| F119L | 76 | PHE | 119 | LEU | **F119L** | 76 | PHE | 136 | PHE |
| F119L | 76 | PHE | 136 | PHE |  |  |  |  |  |
| F119L | 117 | TRP | 119 | LEU |  |  |  |  |  |
| F119L | 117 | TRP | 136 | PHE |  |  |  |  |  |
| N78S | 76 | PHE | 117 | TRP | **N78S** | 76 | PHE | 117 | TRP |
| N78S | 76 | PHE | 119 | PHE | **N78S** | 76 | PHE | 119 | PHE |
| N78S | 76 | PHE | 136 | PHE | **N78S** | 76 | PHE | 136 | PHE |
| N78S | 117 | TRP | 119 | PHE | **N78S** | 117 | TRP | 119 | PHE |
| N78S | 117 | TRP | 136 | PHE |  |  |  |  |  |
| N78S | 119 | PHE | 136 | PHE |  |  |  |  |  |
| Y98H | 76 | PHE | 117 | TRP | **Y98H** | 76 | PHE | 117 | TRP |
| Y98H | 76 | PHE | 119 | PHE | **Y98H** | 76 | PHE | 119 | PHE |
| Y98H | 117 | TRP | 119 | PHE | **Y98H** | 117 | TRP | 119 | PHE |
| Y98H | 117 | TRP | 136 | PHE | **Y98H** | 119 | PHE | 136 | PHE |
| Y98H | 119 | PHE | 136 | PHE |  |  |  |  |  |
