# Supplementary material for: Structural Insights into the Folding Defects of Oncogenic pVHL Lead to Correction of Its Function In Vitro
Source: PLoS One. 2013 Jun 20;8(6):e66333. doi: 10.1371/journal.pone.0066333 (PMC3688787; doi:10.1371/journal.pone.0066333)
Supplement: Table S2 — RMS deviation of C-α in the 4 models of mutants, as compared to the WT structure. (DOCX) [file pone.0066333.s004.docx]

**Table S2.** RMS deviation of C-α in the 4 models of mutants, as compared to the WT structure.

| pVHL missense mutation | Root Mean Square (RMS) | Number of C-α atoms aligned |
| --- | --- | --- |
| F136L | 0.458 | 132 to 132 atoms |
| F119L | 0.258 | 141 to 141 atoms |
| N78S | 0.233 | 136 to 136 atoms |
| Y98H | 0.218 | 136 to 136 atoms |
