# Supplementary material for: Structural Insights into the Folding Defects of Oncogenic pVHL Lead to Correction of Its Function In Vitro
Source: PLoS One. 2013 Jun 20;8(6):e66333. doi: 10.1371/journal.pone.0066333 (PMC3688787; doi:10.1371/journal.pone.0066333)
Supplement: Text S1 — In silico prediction of the effect of oncogenic missense mutations on the structure of pVHL. (DOCX) [file pone.0066333.s005.docx]

**Supplementary**

**SI In silico prediction of the effect of oncogenic missense mutations on the structure of pVHL**

Once the four key structural residues for our analysis were identified, we chose a known oncogenic missense mutation in each of these four residues and examined the effect of these mutations on the structural integrity of pVHL. In doing so we not only elucidated the significance of each of the residues for the wild type pVHL, but also shed light on the folding of pVHL as a whole and the effect of each mutation on the structure and function of the protein.

Three missense mutations in the chosen residues were examined for their effect on folding of pVHL: N78S, F119L and F136L (Figure S1). An additional missense mutation studied was Y98H, which corresponds to a naturally occurring pVHL mutation presumed to be loss-off function since it disrupts the contact of pVHL with the HIF-1α substrate [[1](#_ENREF_1)].

In order to predict the impact of each amino acid substitution on the local and overall stability and folding of pVHL, we produced a model for each mutant protein (via I-TASSER, URL: http://zhanglab.ccmb.med.umich.edu/I-TASSER/). We further evaluated effects of each mutation on the intra- and inter-protein interactions using the Protein Interactions Calculator-PIC (URL: <http://pic.mbu.iisc.ernet.in/>).

The predicted structure of the N78S mutant protein results in a relatively minor influence on the distances between the residues that make up the aromatic tetrahedron (Figure S1), i.e. with the predicted distances at which aromatic-aromatic interactions occur, 4.5-7 Å [[2](#_ENREF_2)]. Residue interaction analysis using PIC revealed that of the four mutations studied, the N78S mutation (along with the Y98H contact mutation) preserves the most aromatic-aromatic interactions that map to the aromatic tetrahedron, as well as the intra-protein hydrophobic interactions, all of which contribute to the core-packing and stability of the protein (see supplementary Table S1).

The structure of the mutant protein carrying the Y98H contact mutation shows an elongation of all faces of the aromatic tetrahedron, in comparison to the WT protein, with distances between the residues at a range of 5.1 to 7.3 Å. Yet, its nullifying effect on pVHL activity is presumably primarily functional as Histidine lacks the hydroxyl group of Tyrosine and is therefore unable to bind the hydroxyproline of HIF-1α.

Both the F119L and F136L mutations affect key residues of the aromatic tetrahedron itself. Once these Phenylalanines are mutated to Leucine, aromaticy is lost, and Leucine, which is less bulky than Phenylalanine, adopts a different orientation relative to the Pyramid and the hydrophobic core of the protein (Figure S1). Furthermore, in these mutant proteins, the aromatic tetrahedron is distorted and is left with only one of its four faces. This observation correlates with the residue interaction analysis obtained via PIC, as there is a significant reduction of predicted aromatic-aromatic and intra-protein hydrophobic interactions caused by these mutations, compared to the WT pVHL (Table S1).

Furthermore, when superimposing all of the mutant pVHL structures we have generated on the structure of the WT pVHL (PDB code 1LM8)[[3](#_ENREF_3)], it is evident that F136L has greatest overall change in pVHL structure and causes the largest exposure of hydrophobic residues of the protein (Figure S1). Particularly noticeable are the difference in orientation and the increase of solvent accessibility of F91, Y175 and Y184 in the mutant F136L protein. These changes have severe effect on the entire residue interaction network (Table S1), suggesting that F136 is a pivotal structural residue of pVHL.

**References**

1. Stebbins CE, Kaelin WG, Jr., Pavletich NP (1999) Structure of the VHL-ElonginC-ElonginB complex: implications for VHL tumor suppressor function. Science 284: 455-461.

2. Burley SK, Petsko GA (1985) Aromatic-aromatic interaction: a mechanism of protein structure stabilization. Science 229: 23-28.

3. Min JH, Yang H, Ivan M, Gertler F, Kaelin WG, Jr., et al. (2002) Structure of an HIF-1alpha -pVHL complex: hydroxyproline recognition in signaling. Science 296: 1886-1889.

# Figure Legends

**Figure S1, related to figure 1.**

**(A-E) Effect of missense mutations on the structure of the aromatic tetrahedron** **in pVHL.**

The aromatic tetrahedron in WT and mutant pVHL proteins (based on PDB code:1lm8), comprising F76, W117, F119 and F136. Mutated residues are highlighted in red.

The impact on the aromatic tetrahedron of each missense mutation shown by the aromatic interaction distances (yellow dashed line). **A.** WT; **B.** F119L; **C.** F136L; **D.** Y98H; **E.** N78S

**(F) Position of the missense pVHL mutations studied.**

Crystal structure of pVHL (gray)(PDB ID code 1lm8) showing the N78, Y98, F119 and F136 residues (red). Missense mutations in these residues cause cancer.

**(G) Exposure of hydrophobic residues in the missense mutant proteins.**

Superimposition of the mutant pVHL structures on the structure of the WT pVHL (blue) (PDB code 1LM8) shown as spheres. Note the overall change in pVHL structure and the resultant exposure of hydrophobic residues (green).

**Figure S2.** Far-UV (**A**) and near-UV (**B**) CD spectra of pVHL, WT (blue) and mutant F136L (red). CD spectra measurements were conducted at 25°C. Protein samples were at final concentration of 3 µM pVHL in 10 mM Tris-HCl (pH 8) and 500 mM NaCl.

**Table S1, related to Figure 1. Output of the Protein Interactions Calculator (PIC).** This table summarizes the PIC output on hydrophobic interactions within 5 angstroms and aromatic-aromatic interactions within 4.5-7 angstroms, relevant to the aromatic tetrahedron.

| **Hydrophobic Interactions within 5 Angstroms** | | | | | **Aromatic-Aromatic Interactions within 4.5 and 7 Angstroms** | | | | |
| --- | --- | --- | --- | --- | --- | --- | --- | --- | --- |
| pVHL type | Position | Residue | Position | Residue | pVHL type | Position | Residue | Position | Residue |
| Wt | 76 | PHE | 117 | TRP | wt | 76 | PHE | 117 | TRP |
| Wt | 76 | PHE | 119 | PHE | wt | 76 | PHE | 119 | PHE |
| Wt | 76 | PHE | 136 | PHE | wt | 76 | PHE | 136 | PHE |
| Wt | 117 | TRP | 119 | PHE | wt | 119 | PHE | 136 | PHE |
| Wt | 117 | TRP | 136 | PHE | wt | 117 | TRP | 119 | PHE |
| Wt | 119 | PHE | 136 | PHE | wt | 119 | PHE | 136 | PHE |
| F136L | 76 | PHE | 117 | TRP | F136L | 76 | PHE | 117 | TRP |
| F136L | 76 | PHE | 119 | PHE | F136L | 76 | PHE | 119 | PHE |
| F136L | 76 | PHE | 136 | LEU | F136L | 117 | TRP | 119 | PHE |
| F136L | 117 | TRP | 119 | PHE |  |  |  |  |  |
| F136L | 117 | TRP | 136 | LEU |  |  |  |  |  |
| F136L | 119 | PHE | 136 | LEU |  |  |  |  |  |
| F119L | 76 | PHE | 117 | TRP | F119L | 76 | PHE | 117 | TRP |
| F119L | 76 | PHE | 119 | LEU | F119L | 76 | PHE | 136 | PHE |
| F119L | 76 | PHE | 136 | PHE |  |  |  |  |  |
| F119L | 117 | TRP | 119 | LEU |  |  |  |  |  |
| F119L | 117 | TRP | 136 | PHE |  |  |  |  |  |
| N78S | 76 | PHE | 117 | TRP | N78S | 76 | PHE | 117 | TRP |
| N78S | 76 | PHE | 119 | PHE | N78S | 76 | PHE | 119 | PHE |
| N78S | 76 | PHE | 136 | PHE | N78S | 76 | PHE | 136 | PHE |
| N78S | 117 | TRP | 119 | PHE | N78S | 117 | TRP | 119 | PHE |
| N78S | 117 | TRP | 136 | PHE |  |  |  |  |  |
| N78S | 119 | PHE | 136 | PHE |  |  |  |  |  |
| Y98H | 76 | PHE | 117 | TRP | Y98H | 76 | PHE | 117 | TRP |
| Y98H | 76 | PHE | 119 | PHE | Y98H | 76 | PHE | 119 | PHE |
| Y98H | 117 | TRP | 119 | PHE | Y98H | 117 | TRP | 119 | PHE |
| Y98H | 117 | TRP | 136 | PHE | Y98H | 119 | PHE | 136 | PHE |
| Y98H | 119 | PHE | 136 | PHE |  |  |  |  |  |

**Table S2, related to Figure 1.** RMS deviation of C-α in the 4 models of mutants, as compared to the WT structure.

| pVHL missense mutation | Root Mean Square (RMS) | Number of C-α atoms aligned |
| --- | --- | --- |
| F136L | 0.458 | 132 to 132 atoms |
| F119L | 0.258 | 141 to 141 atoms |
| N78S | 0.233 | 136 to 136 atoms |
| Y98H | 0.218 | 136 to 136 atoms |
